# Drug-Related Hospital Visits and Admissions Associated with Laboratory or Physiologic Abnormalities—A Systematic-Review

**DOI:** 10.1371/journal.pone.0066803

**Published:** 2013-06-27

**Authors:** Kerry Wilbur, Huda Hazi, Aya El-Bedawi

**Affiliations:** College of Pharmacy, Qatar University, Doha, Qatar; Sapienza University of Rome, Italy

## Abstract

Countless studies have demonstrated that many emergency-room visits and hospital admissions are drug-related and that a significant proportion of these drug-related visits (DRVs) are preventable. It has not been previously studied which DRVs could be prevented through enhanced monitoring of therapy. The objective of the study was to determine the incidence of DRVs attributed to laboratory or physiologic abnormalities. Three authors independently performed comprehensive searches in relevant health care databases using pre-determined search terms. Articles discussing DRV associated with poisoning, substance abuse, or studied among existing in-patient populations were excluded. Study country, year, sample, design, duration, DRV identification method, proportion of DRVs associated with laboratory or physiologic abnormalities and associated medications were extracted. The three authors independently assessed selected relevant articles according to the Strengthening the reporting of observational studies in epidemiology (STROBE) as applicable according to the studies' methodology. The initial literature search yielded a total of 1,524 articles of which 30 articles meeting inclusion criteria and reporting sufficient laboratory or physiologic data were included in the overall analysis. Half employed prospective methodologies, which included both chart review and patient interview; however, the overwhelming majority of identified studies assessed only adverse drug reactions (ADRs) as a drug-related cause for DRV. The mean (range) prevalence of DRVs found in all studies was 15.4% (0.44%–66.7%) of which an association with laboratory or physiologic abnormalities could be attributed to a mean (range) of 29.4% (4.3%–78.1%) of cases. Most laboratory-associated DRVs could be linked to immunosuppressant, antineoplastic, anticoagulant and diabetes therapy, while physiologic-associated DRVs were attributed to cardiovascular therapies and NSAIDs. Significant proportions of laboratory and physiologic abnormalities contribute to DRVs and are consistently linked to specific drugs. These therapies are potential targets for enhanced medication monitoring initiatives to proactively avert potential DRVs.

## Introduction

Drug-related emergency room visits and hospital admissions (DRVs) are a significant contributor to morbidity, mortality and health care costs worldwide. While most documentation of the problem has focused on DRVs attributed to adverse drug reactions (ADRs), few researchers have explored other drug-related problem (DRP) etiologies categorized within the pharmaceutical care nosology, including inappropriate medication selection or dosing; untreated symptoms or disease; drug interactions; and patient non-adherence [1]–[5]. Features of patient populations at-risk for DRVs have been consistently described (the elderly, those with impaired cognition, dependent living situations, renal insufficiency, multiple comorbidities or polypharmacy) as have the most common offending therapies (antiplatelets, anticoagulants, non-steroidal anti-inflammatory drugs (NSAIDS), diuretics, angiotensin converting enzyme (ACE) inhibitors, opioids, and diabetes treatments) [6]–[8].

Several broad recommendations for the reduction of preventable DRVs have been proposed such as improving communication between acute and ambulatory health care providers when patients transition between care settings; conducting regular review of prescription medications to avoid therapeutic duplication and to discontinue unnecessary drugs; advising patients to frequent one community pharmacy and to discuss self-selection of over-the-counter (OTC) and herbal therapy with a pharmacist or physician [9]–[11]. Enhanced patient monitoring is also frequently suggested. Baseline and follow-up assessment of renal function in populations at-risk is most often cited, but improving the monitoring of other specific laboratory values according to the prescribed therapy (e.g. INR for anticoagulated patients, potassium for diuretic-treated patients) is also advocated. Unfortunately, low adherence to enhanced laboratory monitoring has been demonstrated even when straightforward protocols are devised [12]–[14]. Early detection of DRPs does not always require blood testing; certain unfavourable medication responses manifesting clinically may be recognized by straightforward patient assessment. Simple vital sign evaluation is efficient and non-invasive and therefore has potential for greater drug monitoring adherence. Altered body physiology leading to harmful conditions and attributed to medication may be generally grouped within the broader context of assignment of ADR-associated DRVs, but their differentiation is important as distinct preventative measures may be considered [15].

Our study objective is to estimate what proportion of total DRVs are associated with laboratory or physiologic abnormalities and therefore potentially be prevented with augmented monitoring systems.

## Methods

### Searching

Three authors (KW, HH, AE) independently performed comprehensive searches in relevant health care databases: PubMed (1966-November Week 1 2011); Embase (1947-November Week 1 2011); EBM Reviews – Cochrane Central Register of Controlled Trials (March 1996 to 3^rd^ Quarter 2011); Web of Science® (1960-November Week 1 2011); Scopus® (1996-November Week 1 2011); Science Direct® (1995-November Week 1 2011); and Latin American Caribbean Health Sciences Literature (1982–2011). Predetermined search terms included key words and phrases: “medication-related” or “drug-related”; “hospitalization”; “emergency department”; “visit” or “admission”. No language restrictions were applied. Search strategies were modified to accommodate the controlled vocabulary in these databases. References of retrieved articles were also hand-searched.

Abstracts of unpublished studies were identified by electronic searching of available databases: International Pharmaceutical Abstracts (1970-November Week 1, 2011) and Cumulative Index to Nursing and Allied Health Literature (1982-November week 1, 2011) as well as by hand-searching International Society of Pharmaceutical Outcomes Research, American College of Clinical Pharmacy, American Society of Health-System Pharmacists, Canadian Society of Hospital Pharmacy, European Society of Clinical Pharmacy conference proceedings. Pre-determined search terms were also applied to a general internet search using Google Scholar.

### Study Selection and Characteristics

The titles and abstracts of articles identified by the search were screened for potential relevance. The duplicate article titles identified among the searches by the three authors were eliminated. Full-text of potentially relevant studies were retrieved and considered eligible for inclusion according to pre-determined selection criteria: 1) evaluation of drug-related presentation to an ED for care; and/or 2) evaluation of a drug-related admission to an inpatient setting; and 3) data reported in sufficient detail to identify drug-related ED visit or hospitalization associated with abnormal laboratory value/s or physiologic findings. Adverse physiologic events were identified according to study documentation of specific clinical findings: 1) elevated or decreased systolic blood pressure; 2) elevated or decreased heart rate; 3) elevated or decreased respiratory rate; 4) elevated temperature or; 5) overt bleeding event [15]. Studies of both adult and pediatric populations were included. Articles were excluded if they: 1) described ED visit or hospitalization due to illicit drug use or abuse or intentional overdose; 2) described drug-related visits to ambulatory or primary care settings; 3) described inpatient detection of drug-related problems; or 4) were narrative reviews or commentaries. Disagreements about inclusion were resolved in author consensus meetings.

### Validity Assessment

The three authors independently assessed selected relevant articles according to the Strengthening the reporting of observational studies in epidemiology (STROBE) guide as applicable according to the studies' methodology to guide data extraction [16]. The methods for identification and criteria for subsequent characterization of a drug-related cause for emergency visit or hospitalization; methods used to establish causal relationship between the medication and observed outcome; tools for assessing the preventability and the severity of the DRV were also assessed to determine study quality and were graded as yes, no, unclear, or not reported [17].

### Data Abstraction

A standardized data extraction form was developed according to the studies' variables of interest: year of publication; country of original; study setting, design; population; methods of data collection and profession of data collectors; means for categorizing potential drug-related visits and admissions; means for characterizing preventability of drug-related visits and admissions; number and nature of drug-related laboratory or physiologic abnormalities; and finally the medications associated with these. Final inclusion and exclusion decisions were then made by author consensus.

### Qualitative Data Synthesis

The methodological heterogeneity across studies including identification and classification of DRPs precluded rigorous quantitative assessment (meta-analysis) and so the study results are described and evaluated qualitatively.

## Results

### Flow of Included Studies

**Figure 1:**
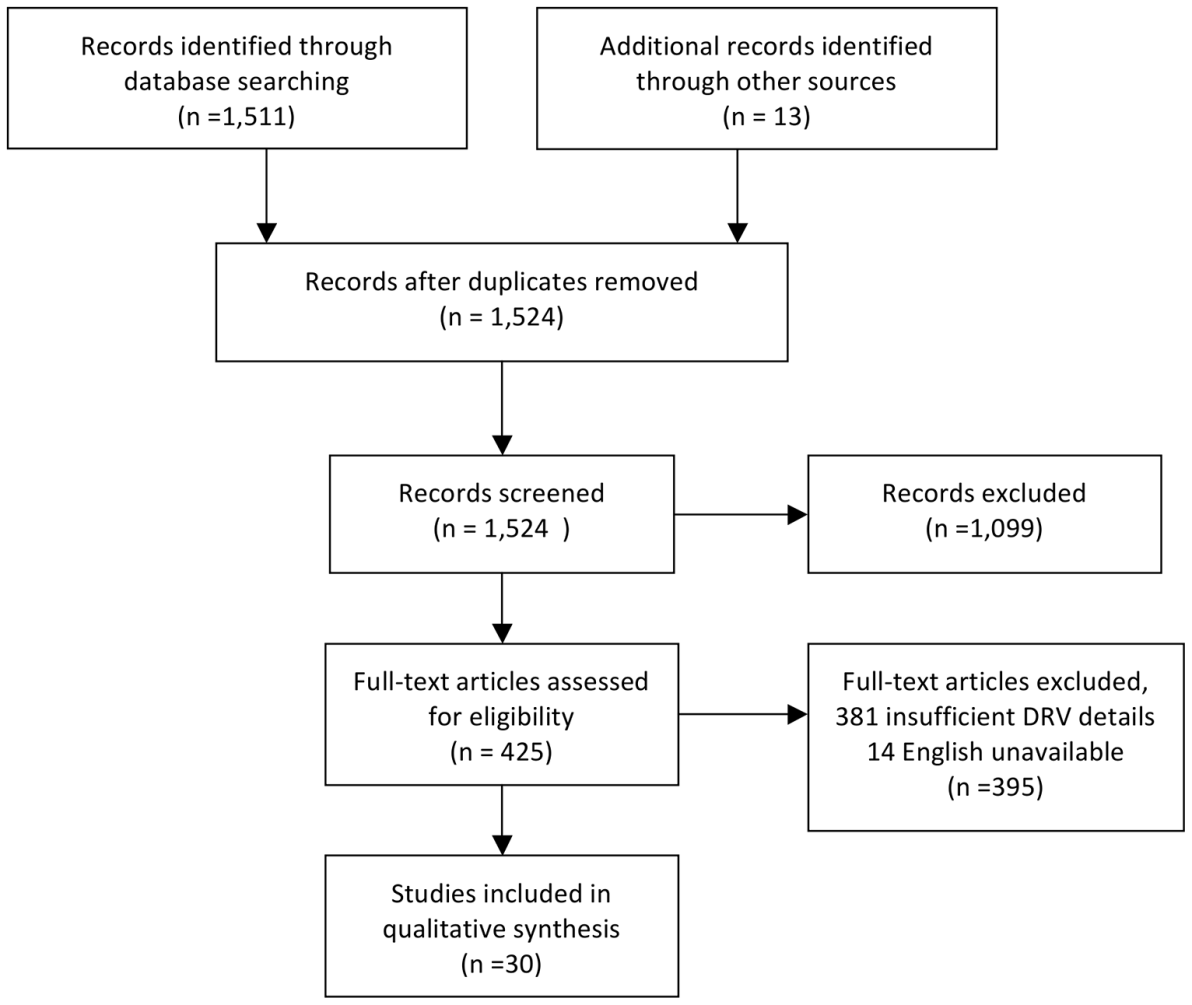
Flow chart of the process of identifying selected articles. This figure describes the outcomes of our outlined search strategy at various stages of the systematic review. The three authors conducted structured searches in several databases, which yielded 1,524 citations. When these titles were screened, 270 duplicate articles were identified and removed. Of the remaining titles, only 425 met our main inclusion criteria: 256 were excluded as they described illicit drug use or abuse-related DRV; 187 were DRV study in ambulatory or primary care populations; 114 were DRV study in hospitalized populations; and 272 were reviews, commentaries, editorials or letters. Subsequently 381 full text articles assessed reported data in insufficient detail to ascertain if documented DRVs could be associated with laboratory or physiologic abnormality and 14 abstracts were not available in full-text English. The remaining 30 studies were included in the review.

The initial literature search yielded a total of 1,524 articles (Figure 1). After reviewing the titles and abstracts, 1,099 articles were excluded. These articles included duplicate titles (270), description of DRVs due to illicit drug use or abuse (256); reports of DRVs to ambulatory or primary care settings (187); research of inpatient detection of DRPs (114); or were narrative reviews, commentaries, editorials or letters (272). Four-hundred and twenty-five studies remained for full text review, but despite an English abstract, 14 were not available in English and 408 did not provide sufficient details to determine laboratory or physiologic abnormalities among the DRVs or admissions recorded. We included 30 articles in the review.

### Study Characteristics

Study characteristics of the included articles are described in Table 1 [18]–[47]. A total number of 98,138,737 patient admissions were evaluated in the included studies. The majority was conducted among adult populations only and 10 specifically assessed geriatric-DRVs. Only 1 study was performed in pediatrics. The studies were conducted in health care settings throughout the world, but predominantly in Europe, and spanned over a wide range of evaluation time periods (2 weeks to 26 years). Seven studies examined drug-related ED visits alone, while 13 involved drug-related hospital admission to particular patient care units, usually internal medicine or related sub-specialty ward/s. Half of the studies employed prospective methodologies that included patient interview complemented by medical chart review. Three reports estimated DRVs according to retrospective study of national hospitalization databases.

**Table 1:** Characteristics of included studies.

Study	Country	Participants	DRV	Study Design	Study Method	Duration	DRPs Evaluated	DRVs attributed to DRPs, n (%)	Laboratory- and/or Physiologic-Related DRV, n (%)
Foisy 2000	Canada	Adult with HIV	Hospital	R	CR	1 Y pre- and post- initiation of HAART	ADR, DI associated with HAART	4.4% (19/436) pre-HAART therapy 7.7% (25/323) post-HAART therapy	89% (17/19) laboratory pre-therapy 64%(16/25) laboratory post-therapy
Green 2000	UK	Adult	ED	P	CR & PI	Unclear	ADR	7.5% (15/200)	33% (5/15) laboratory 20% (3/15) physiologic
Chan 2001	Australia	Adult ≥75 Y	ED	P	CR & PI	2 M	All DRPs	30.4% (73/350)	11% (8/73) laboratory 37% (27/73) physiologic
Hohl 2001	Canada	Adult >65 Y	ED	R	CR	1 Y	ADR, DI	10.6% (30/282)	23.3% (7/30) laboratory 16.6% (7/30) physiologic
Wasserfallen 2001	Switzerland	Adult	ED	P	CR & PI	6 M	ADR	7.2% (229/3,195)	10.9% (25/229) laboratory 44.5% (102/229) physiologic
McDonnell 2002	US	Adult	Hospital	R	CR	11 M	ADR	62.3% (96/154) *preventable ADR	69.7% (67/96) laboratory+physiologic
Mjorndal 2002	Sweden	Adult	IMD, Cardio	P	CR & PI	9 M	ADR	18.1% (82/452)	19.5% (16/82) laboratory 15.8% (13/82) physiologic
Howard 2003	UK	Adult	Hospital	P	CR & PI	6 M	All DRP	6.5% (265/4,093)	18.1% (48/265) laboratory 4.2% (11/265) physiologic
Juntti-Patinen 2006	Finland	Adult >60 Y	ED	P	CR & PI	6 M	ADR	1.4% (102/7,113)	18.6% (19/102) laboratory2.9% (3/102) physiologic
Von Euler 2006	Sweden	Adult	IMD	R	CR	3 W	ADR	11% (18/168)	33.3% (6/18) laboratory27.7% (5/18) physiologic
Patel 2007	UK	Adult	Hospital	R	National database	7 Y	ADR	0.5% (68,971/88,822,005)	8.3% (5,752/68.971) laboratory
Rivkin 2007	US	Adult	MICU	P	CR & PI	6 M	ADR	7.5% (21/281)	19% (4/21) laboratory 4.7% (1/21) physiologic
Alexapoulou 2008	Greece	Adult	IMD	P	CR & PI	6 M	ADR	12.7% (70/548)	42.8% (30/70) laboratory 40% (28/70) physiologic
Franchesci 2008	Italy	Adult ≥65 Y	Hospital	P	CR & PI	13 M	ADR	5.8% (102/1,756)	12.7% (13/102)laboratory 2.7% (13/102) physiologic
Hopf 2008	Scotland	Adult	Hospital	P	CR & PI	15 D	ADR	1.3% (30/2,371)	30% (9/130) laboratory 40% (12/30) physiologic
Bravar 2009	Slovenia	Adult	ED, IMD specialty depts	R	CR	1 Y	ADR	5.8% (30/520)	46.7% (14/30) laboratory 53.3% (16/30) physiologic
Rogers 2009	UK	Adult ≥65 Y	IMD	P	CR & PI	3×1 M	All	5.4% (26/485) and 61.5% (16/26) considered reventable	31.25% (5/16) laboratory 37.5% (6/16) physiologic
Wawruch 2009	Slovakia	Adult ≥65 Y	IMD	R	CR	16 M	ADR	7.8% (47/600)	23.4% (11/47) laboratory 40.4% (19/47) physiologic
Davies 2010	UK	Adult	Hospital	R	CR	Unclear	ADR	16.9% (73/403)	35.6% (26/73) laboratory 20.5% (15/73) physiologic
Fokter 2010	Slovenia	Adult	Hospital	R	CR	1 Y	DI, ADR	51.4% (166/323) DI 6.2% (20/323) ADR	7.4% (6/166+8/20) laboratory 21.3% (28/166+12/20) physiologic
Hartholt 2010	The Netherlands	Adult >60 Y	Hospital	R	National database	26 Y	ADR	247,638 DRV total admissions not reported	4.5% (10,641/247,638) laboratory 2.5% (6,206/247,638) physiologic
Rodenburg 2010	The Netherlands	Adult	Hospital	R	National database	5 Y	ADR	0.44% 41,260/9,287,162	15.5% (6,405/41,260) laboratory 13.6% (5,632/41,260) physiologic
Somers 2010	Belgium	Elderly Adult	Geriatric ward	P	CR & PI	3×1 M	All	21% (23/110)	20% (5/25) laboratory 28% (7/25) physiologic
Gallagher 2011	UK	Pediatric	ED, Hospital	P	CR & PI	2 W	ADR	1.1% ED (9/847) 3.2% Hospital (27/847)	44.4% (12/27) laboratory 3.7% (1/27) physiologic
Hellstrom 2011	Sweden	Adult	ED, IMD	P	CR & PI	4×1 M	All DRPs	(8/86) 40.9%	37.5% (3/8) laboratory 37.5% (3/8) physiologic
Hofer-Dueckelmann 2011	Germany	Adult	Cardio, Nephro, GI wards	P	CR & PI	2×3 M	ADR	7.6% (242/3,190)	78% (189/242) laboratory 7.8% (19/242) physiologic
Marcum 2011	US	Adult ≥65 Y	Hospital	R	CR	2Y	ADR	10% (68/678)	29.4% (20/68) laboratory 25% (17/68) physiologic
Miranda 2011	Brazil	Adult	Oncology ward	R	CR	8 M	ADR, DI	13.1% (39/298) (33 ADR+6 DI)	51.3% (20/39) laboratory 15.4% (6/39) physiologic
Noize 2011	France	Adult	IMD specialty depts	R	CR	1 Y	ADR (hyperkalemia)	66.7% (112/168)	
Varallo 2011	Brazil	Adult >60 Y	IMD	P	CR & PI	5 M	ADR	42.9% (60/140)	8.3% (5/60) laboratory+physiologic
MEAN (Range)								15.4% (0.44–66.7%)	50.7% (6.8%–100%)

ADR: adverse drug reaction; Cardio: cardiology; CR: chart review; Depts: departments; DRP: drug-related problem; DRV: drug-related visit; ED: emergency department; GI: gastrointestinal; HAART: highly active antiretroviral therapy; IMD: internal medicine; M: months; MICU: medical intensive care unit; Nephro: nephrology; P: prospective; PI: patient interview; R: retrospective; Y: years.

Adverse drug reaction-associated DRVs was the predominant DRP evaluated (25/30) and combined with drug interaction-associated DRVs in 4 of these studies. Five studies included all DRPs when determining DRV etiology (Table 1). The WHO definition for ADR (14/25) or a comparable one (5/25) was used in the studies evaluating ADR-associated DRVs, but 2 did not explicitly offer an ADR description. The 3 studies using patient registries screened ADR-related DRVs according to recorded ICD-9CM coding systems. All studies evaluating drug interactions described their tertiary and secondary resources. When investigators considered all DRPs in determining DRVs (5/30), recognized categories outlined by Hepler and Strand or Hallas were employed [1], [48].

Nearly three quarters of the studies (25/30) mentioned a causality assessment of the suspected DRV, of which the Naranjo algorithm (9/30) and WHO algorithm (8/30) were the most frequently used tools. Few studies (10/30) performed an assessment of the severity of patient outcome resulting from DRVs and only half of these descriptions were derived from a cited reference. Eighteen studies did not report assessment of the preventability of the DRVs they identified but 12 used recognized tools, such as Hallas, Schumock and Thornton, and Guruwitz [48]–[50].

Thirteen studies reported that a clinician was involved in the initial patient data screening and identification of the DRVs and followed a secondary process of patient data evaluation to subsequently validate the classification [21], [24], [25], [29], [30], [32]–[34], [41]–[45]. In these reports, the study team was often multidisciplinary, and may have included physicians, pharmacists, or nurses. Seven additional studies also described initial clinician determination of DRVs, but did not report additional independent case adjudication [18], [19], [26], [27], [36], [37], [40]. Ten studies did not provide information about who identified the DRVs [20], [22], [23], [28], [31], [35], [38], [39], [46], [47].

### Data Synthesis

**Figure 2:**
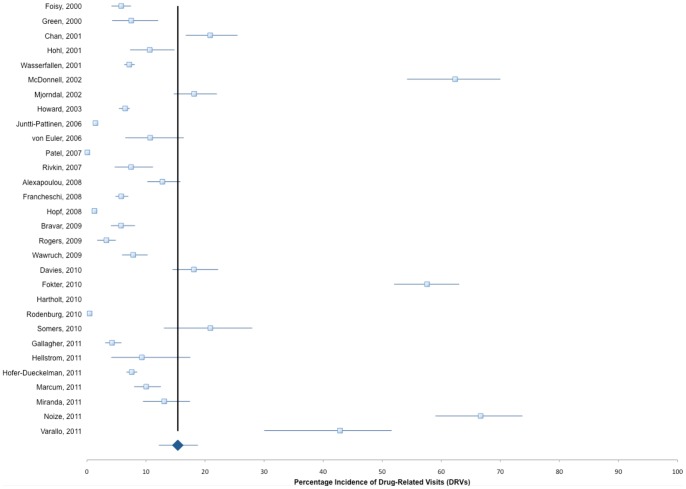
Drug-Related Visits, overall pooled mean incidence and 95% CI.

**Figure 3:**
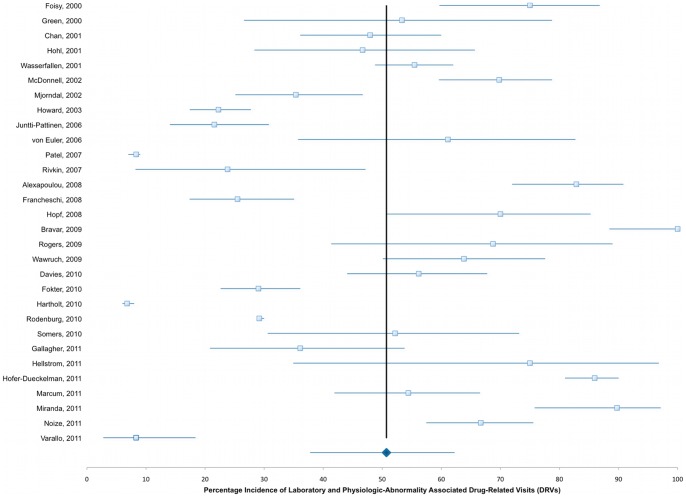
Laboratory and Physiologic-Abnormality Associated DRV, overall pooled mean incidence and 95% CI.

**Figure 4:**
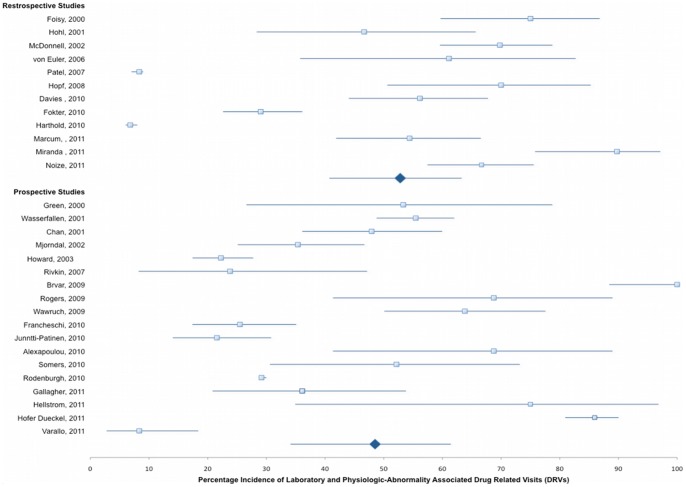
Laboratory and Physiologic-Abnormality Associated DRV According to Study Methodology, mean incidence and 95% CI.

**Figure 5:**
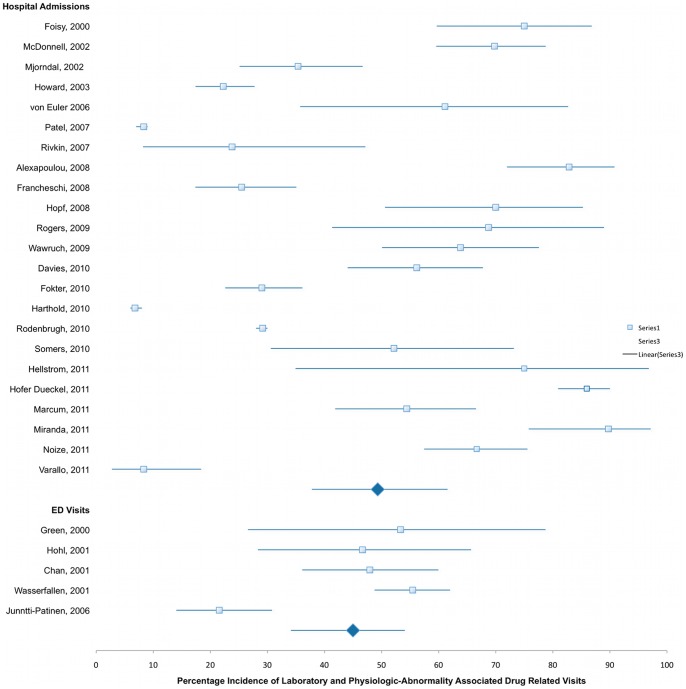
Laboratory and Physiologic-Abnormality Associated DRV According to Patient Setting, mean incidence and 95% CI.

**Figure 6:**
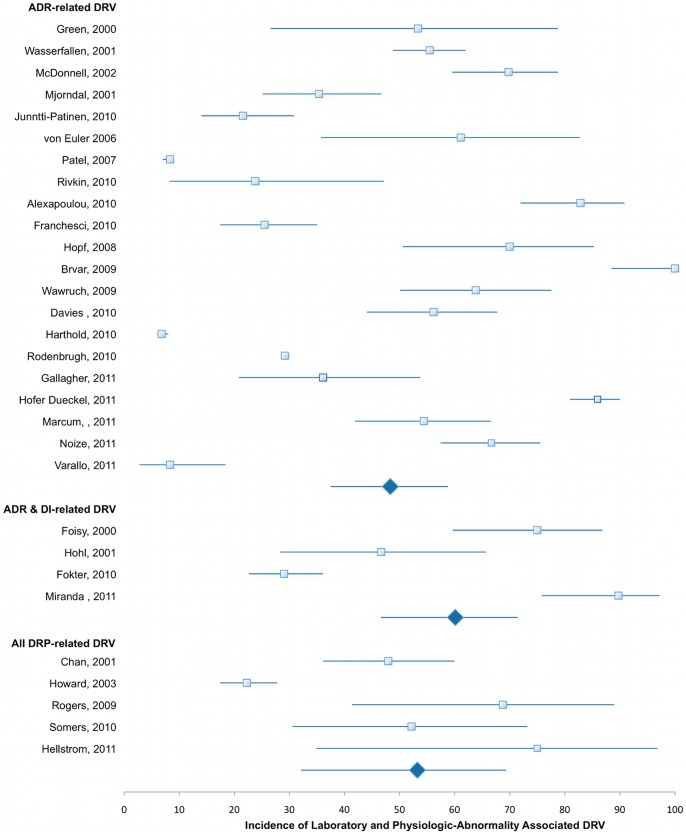
Laboratory and Physiologic-Abnormality Associated DRV According to Drug-Related Problem (DRP), mean incidence and 95% CI.

The mean (range) prevalence of DRVs found in these studies was 15.4% (0.44%–66.7%) (Figure 2). In our pooled analysis, the overall mean (range) proportion of these DRVs that could be attributed to insufficient patient medication monitoring was 50.7% (6.8%–100%) (Figure 3). We could identify laboratory abnormalities in a mean of 29.4% (range 4.3%–78.1%) and findings of adverse physiologic events in a mean of 23.3% (range 2.5%–53.3%) contributing to the overall rate of DRVs as recorded by the investigators. Even if studies sampling subjects from national or hospital databases are censored and the evaluated patient admissions in our analysis drops considerably from 98,138,737 to 29,570, mean estimated prevalence of overall DRVs, laboratory- and physiologic-related DRVs are not considerably altered (16.5%, 31.9%, and 24.5%, respectively). There is also no appreciable difference when retrospective (52.8%) and prospective (49.3%) studies are compared (Figure 4). When studies evaluating laboratory- and physiologic-related DRV to the ED department alone are compared to those only evaluating DRVs leading to hospital admission, the incidence is somewhat higher (49.3% vs 44.9%, respectively, Figure 5). Observed mean estimates of the incidence of laboratory- and physiologic-related DRVs varied according to length of evaluation reported by (<3 months 55.8%; 3–6 months 46.9%; 7–11 months, 57.9%; 1–5 years, 43.2%; and >5 years, 7.67%). When all DRP-related admissions are considered, the overall incidence of DRV is 53.2% compared to those studies evaluating only one DRP (ADRs, 48.3%) or two DRPs (ADRs and DIs, 60.1%) contributing to the DRV (Figure 6).

The most common laboratory-related DRVs were abnormalities in electrolytes (hyponatremia, hyper- and hypokalemia); blood dyscrasias (anemia, neutropenia); metabolic disturbances (hyper- and hypoglycemia); and acute renal failure. Overt bleeding events (upper gastrointestinal bleeding, melena), hypotension and bradycardia were most frequently identified adverse physiologic events. Laboratory-associated DRVs were most linked to anticoagulant, antiplatelet, diabetes and immunosuppressant drugs, whereby cardiovascular and NSAID analgesic therapeutic categories were ranked highest for physiologic-associated DRVs. Culprit drug therapy identified by investigators included loop diuretics, ACE inhibitors, aspirin, wafarin, thiazide diuretics, digoxin, beta blockers, sulphonylureas, and corticosteroids. Laboratory assessment detected values outside recognized therapeutic ranges in patients anticoagulated (elevated INR, n = 64), receiving digoxin (n = 29), antiepileptic therapy (n = 23) and lithium (n = 5).

## Discussion

This is the first systematic review of DRVs attributed to complications associated with abnormalities in laboratory values and adverse physiologic events. Prior work has also sought to quantify the prevalence of monitoring problems contributing to preventable hospital admissions and found median (range) rate of 22.2% (0–31.3%) across 5 papers [6]. In that study, the characterization of a “monitoring problem” was not overtly defined and presumably relied upon the interpretation of the original authors of the studies in question. Our review is more broad, in that we made an independent determination of laboratory or physiologic abnormalities according to the detailed DRV data reported by the investigators and considered studies which did not necessarily ascribe preventability to its identified DRVs.

We found that on average half of identified DRVs were associated with patient morbidity that researchers linked with an irregularity in a monitored parameter. Apparent evidence of these laboratory and physiologic abnormalities contributing to DRV is consistently reported across the studies we included in our analysis, but incidences are highly variable (4.3%–100%). The broad range is not dissimilar to study findings of DRV prevalence in general and it is known that methodological factors impact observed rates [51]. Our review similarly includes diverse study settings and populations with considerations of all hospital admissions as well as acute admissions to specific patient care areas or ED visits alone. Just as identified by Leendertse et al, we too found that medical chart screening yielded higher prevalence of DRVs and subsequent linkage to laboratory or physiologic abnormality than in our three included studies of retrospective review of databases [51]. However, retrospective chart review usually reported only slightly higher rates than the prospective chart reviews in our study. Prospective data collection complemented by patient interview may yield more conservative estimates as the subject is available to inform or refute existence of a DRV whereas appropriate characterization validation is unavailable in retrospective studies.

We examined investigator methods for detecting a DRV, how causality was established between the untoward event and offending drug therapy, as well as its preventability and severity. Despite global interest in comprehensive evaluation of DRVs that may be associated with any category of DRPs, a valid assessment tool is lacking. Conversely, a number do exist specifically for identification of suspected ADRs experienced by patients and were employed by most researchers of the studies we included. We found most studies applied a recognized causality assessment, but few reported the severity or estimated preventability of the DRVs. The ability to classify DRVs by preventability provides a mechanism for clinicians to implement specific interventions and subsequently evaluate their impact on patients and health systems. Having said this, our review inherently emphasizes DRVs associated with patient parameters whereby augmented monitoring strategies could identify and address potential concerns before the patient was faced with an ED visit or hospital admission. The validity and reliability of DRV detection as reported in the included studies is weak. The majority seemed to rely on a single initial screening phase without further independent review. Professional characteristics of the reviewer must also be considered when exploring reliability in DRV detection. It has been previously demonstrated that physicians overlook DRPs as a cause for emergency department visits [52],[53].

Adverse drug reactions were the predominant DRP investigated in our included studies. The ADR definition most often employed was that of the WHO and defined as “any response to a drug which is noxious and unintended occurring at human doses for prophylaxis, diagnosis and therapy” [54]. A laboratory or physiologic abnormality may arise from an exaggerated, but unintended response (e.g. bradycardia with beta-blockers) or an unexpected adverse reaction unrelated to conventional pharmacology (e.g. agranulocytosis with chlorpromazine). It is worthwhile for future DRVs studies to incorporate alternate DRPs as sources of laboratory and physiologic abnormalities as these can occur from problems with medication adherence (e.g. low INR reported in anticoagulated patient admitted for stroke); drug interaction (e.g. subtherapeutic phenytoin value reported in patient receiving concomitant antibiotic and admitted with seizure); or inappropriate prescribing (e.g. increasing ACE inhibitor dose in the face of hyperkalemia) [55]. Efforts to ameliorate deficiencies in laboratory monitoring of specific drug therapies or of specific patients have been described with few successes. Suboptimal laboratory monitoring occurs under conditions when there is both failure to conduct indicated tests at baseline or at appropriate follow-up intervals and inadequate response when results are reported [56].Patients' prescribed therapy attributed to findings of inappropriate laboratory parameters contributing to DRVs in our included studies are medications already known to possess risks of organ-system toxicities or imbalances to electrolytes and metabolic factors. While monitoring plans have already been outlined for a number of these therapies, a host of practical implementation barriers have been identified such as unclear assignment of responsibility when multiple physicians are involved in care; lack of alerts/reminders; physician fatigue and inaction due to indiscriminant computer alters/reminders; and patient non-adherence to instructions, to name just a few [57]–[59]. Team-based and interdisciplinary approaches are needed to promote appropriate medication monitoring. As highly accessible qualified health care providers, pharmacists can assume shared responsibility with prescribers to ensure appropriate laboratory assessments are conducted and reviewed. When electronic clinical decision support is in place, pharmacists improve patient care and safety by reducing potentially inappropriate prescribed therapy [60]. Studies have also shown that such pharmacist-led laboratory alert prompting prescriber medication review can be cost-effective [61]. When such patient data are not readily available, it is reasonable to consider acquisition of relevant laboratory information or physiologic assessments mandatory as a required element for safe and responsible provision of therapy, not unlike determination of a patient's drug allergy status prior to prescription processing and dispensing [62],[63].

Our review confirms that lack of adequate laboratory monitoring contributes to patient morbidity; however our evaluation of the occurrence of adverse physiologic events is a concept both not well defined and perhaps consequently not previously explored. Physical exam is a systematic process for investigating the body and its function. Physical assessment skills necessary for health professionals to conduct complete and accurate patient evaluations have largely remained in the domain of physicians, nurses, and even physiotherapists. Pharmacist roles and responsibilities to ensure the optimal outcomes from the use of medication are patient-centered and expanding in scope, which includes developing skills in percussion, palpation and auscultation [64],[65]. Combined with customary verbal patient information gathering and inspection skills, pharmacists are increasingly equipped to recognize adverse physiologic effects of drugs through vital sign assessment and other straightforward physical exams. Observation and measurement of abnormal vital signs can be early indicators of potential or actual DRPs and is an area of medication monitoring to be explored in study of DRV prevention.

A number of limitations to our review warrant discussion. Information for our analysis was derived from the published reports and we did not contact authors of screened studies for further data, which may diminish how representative our review is in the context of other available literature. Furthermore, yield from our search for any preliminary data (e.g. published conference proceedings) and non-English language sources was negligible, underscoring just some of the effects of publication bias which we did not attempt to estimate or overcome with corrective techniques (e.g. modeling, fail safe N). We did not explicitly use “adverse drug reaction” in our search strategy and although most of our included studies assessed this particular DRV etiology, it is possible we could have missed some evaluations of DRV due to this or any other singular and specifically described DRP.

There has been little definitive work regarding adverse physiologic events and in the past might be incorporated within the context of a medication ADRs. We defined these events according to one prior report that only encompassed abnormal vital signs to which we added episodes of major or minor bleeding. Our working definition likely underestimated the prevalence of physiologic adverse events. For example, a number of studies included in our review reported exacerbations of heart failure as a DRV. If the accompanying documentation did not specifically refer to altered respiratory status (a vital sign), we did not characterize that patient's DRV as an adverse physiologic event. It is possible that future interpretations may accommodate other patient outcomes easily screened through simple physical assessment or patient interview, such as altered level of consciousness or peripheral edema. Finally, little can be concluded from our review regarding the specific nature of DRVs in children as the majority of included study populations were adults.

## Conclusions

This review demonstrates laboratory abnormalities and adverse physiologic events represent a large proportion of documented drug-related emergency room visits and hospital admissions. Current efforts to ameliorate deficiencies in patient medication monitoring in this regard require augmentation with additional multidisciplinary approaches and subsequent evaluation to determine if interventions actually impact patient outcomes and prevent DRVs. Further work is also required to explore the potential for early detection of adverse physiologic effects of drugs through patient physical assessment by primary health care providers, namely pharmacists.
